# Automated detection of pulmonary embolism from CT-angiograms using deep learning

**DOI:** 10.1186/s12880-022-00763-z

**Published:** 2022-03-14

**Authors:** Heidi Huhtanen, Mikko Nyman, Tarek Mohsen, Arho Virkki, Antti Karlsson, Jussi Hirvonen

**Affiliations:** 1 Department of Radiology, University of Turku and Turku University Hospital, Turku, Finland; 2 Reaktor Innovations Oy, Helsinki, Finland; 3 Auria Clinical Informatics, Turku University Hospital, Turku, Finland; 4 Department of Mathematics and Statistics, University of Turku, Turku, Finland; 5 Auria Biobank, Turku University Hospital, University of Turku, Turku, Finland

**Keywords:** Artificial intelligence, Emergency radiology, Pulmonary embolism, Deep learning, Automated detection

## Abstract

**Background:**

The aim of this study was to develop and evaluate a deep neural network model in the automated detection of pulmonary embolism (PE) from computed tomography pulmonary angiograms (CTPAs) using only weakly labelled training data.

**Methods:**

We developed a deep neural network model consisting of two parts: a convolutional neural network architecture called InceptionResNet V2 and a long-short term memory network to process whole CTPA stacks as sequences of slices. Two versions of the model were created using either chest X-rays (Model A) or natural images (Model B) as pre-training data. We retrospectively collected 600 CTPAs to use in training and validation and 200 CTPAs to use in testing. CTPAs were annotated only with binary labels on both stack- and slice-based levels. Performance of the models was evaluated with ROC and precision–recall curves, specificity, sensitivity, accuracy, as well as positive and negative predictive values.

**Results:**

Both models performed well on both stack- and slice-based levels. On the stack-based level, Model A reached specificity and sensitivity of 93.5% and 86.6%, respectively, outperforming Model B slightly (specificity 90.7% and sensitivity 83.5%). However, the difference between their ROC AUC scores was not statistically significant (0.94 vs 0.91, *p* = 0.07).

**Conclusions:**

We show that a deep learning model trained with a relatively small, weakly annotated dataset can achieve excellent performance results in detecting PE from CTPAs.

**Supplementary Information:**

The online version contains supplementary material available at 10.1186/s12880-022-00763-z.

## Background

Acute pulmonary embolism (PE) is a life-threatening condition and has an estimated incidence of 60 per 100,000 [1–3]. Symptoms of PE are often non-specific, e.g., dyspnoea and chest pain, and clinical diagnosis can be challenging. Computed tomography pulmonary angiography (CTPA) has become the golden standard in diagnosing PE [4], but accurate interpretation of CTPAs requires experience and time. However, the majority of CTPAs are negative, which might be attributed to clinicians not routinely using pre-test probability or rule-out criteria, and to a generally increasing utilization of emergency CT imaging [5, 6]. In the published literature, the yield of positive studies varies from less than 10 to 20–30% [5, 7–9]. Because increasing workload and fatigue related to after-hours work may cause more diagnostic errors in emergency radiology [10–12], an automated PE detection system could aid radiologists to avoid mistakes and prioritize the reading order of studies to ensure rapid evaluation of PE positive cases.

The emergence of deep learning (DL), a subfield of artificial intelligence (AI), has increased interest in automatized detection and diagnostic tools in radiology [13]. Before that, multiple different computer-aided detection (CAD) models for diagnosing PE had been developed using manually encoded methods like segmentation, detection of low-attenuated areas, and/or feature analysis [14, 15]. Use of CAD as a concurrent reader has been shown to increase reader sensitivity [16], but high yield of false positives (FPs) has remained as a major drawback [17]. FPs can cause frustration to radiologists, but they can also increase the risk for false diagnoses due to automation bias—the tendency for humans to favor machine-made decision [18, 19]. Implementation of DL to PE detection has led to models generating fewer FPs without reducing sensitivity [20, 21]. The Radiological Society of North America (RSNA) chose PE detection as its AI challenge in 2020, and later published a public dataset of 12 195 annotated CTPA studies to encourage the development of PE detection models [22].

One limitation of the previous CAD systems and some of the newer DL-based models is the requirement for densely annotated training data, where each distinct embolus is marked or segmented manually. Weikert et al. tested a prototype commercial model (Aidoc Medical, Tel Aviv, Israel) based on fast region-based convolutional neural network (CNN) and trained with 28,000 segmented CTPAs. The model achieved sensitivity and specificity of 92.7% and 95.5%, respectively [23]. Buls et al. also tested the model (version 1.3) and achieved a similar specificity of 95% but a lower sensitivity of 73% [24]. However, creating an annotated training set of this magnitude for own model development may not be feasible for single hospitals or research teams. Using small datasets can lead to overfitting, meaning the model learns the training data too well and generalizes poorly to new data. Overfitting can be tackled to some degree with different regularization techniques, like dropout and early stopping [25].

Weakly supervised learning with sparser annotations is another way to reduce the manual annotation work without reducing the training set size too much. Rajan et al. proposed a sparse annotation method where emboli contours were drawn only for every 10 mm of CTPA slices, reducing the required manual work considerably, yet achieving an area under the receiving operating characteristics (ROC AUC) of 0.78 [26]. Feng et al. proposed a weakly supervised 3D CNN model for lung nodule segmentation and detection requiring only single-coordinate nodule annotations, which could also be applied to PE detection in a similar fashion [27]. Huang et al. assigned only binary labels of PE present or PE absent to CT slices, and their model achieved sensitivity and specificity of 73% and 82%, respectively [28]. Recent advancements with generative adversarial networks (GAN) have enabled creating realistic synthetic data to increase the training set as well as completely unsupervised training of anomaly detection models, although these techniques have not been studied in PE detection yet [29–31].

We sought to develop a neural network model to aid in detecting PE from CTPAs. The model could be used to reduce mistakes or to pre-screen studies to prioritize reading order. Our aim was to study whether training a well-performing model is possible even with limited resources for collecting and annotating data. We used weakly labelled data with slice-based annotations instead of annotations for each distinct embolus. We also used a relatively small training set consisting of only 600 CTPAs, which is less than in many previous studies [23, 26, 28]. To handle the volumetric nature of CT images, we used both 2D convolutional neural network (CNN) to analyze individual slices, and long-short term memory network (LSTM) to process scans as sequences of slices, a combination previously shown to be useful with weakly annotated CT data [32].

## Materials and methods

For this retrospective cohort study, we obtained permission from The Hospital District of Southwest Finland. Waiver for written patient consent was not sought from the institutional review board (IRB, called the Ethics Committee of The Hospital District of Southwest Finland), because it is not required by the national legislature for retrospective studies of existing data. The study was conducted in accordance with the Declaration of Helsinki.

### Data selection and labelling

The PACS records of Turku University Hospital were searched for all CTPA examinations between January 2016 and October 2018. Preliminary classification of the CTPAs was done automatically by assessing whether the patient had an ICD-10 code consistent with PE (I26) or not in the electronic health record. This first classification was used only to collect enough positive cases among the large number of negative cases; subsequently, all final labels in both classes were manually assigned. First, a randomly sampled cohort was selected from the image archive which included data from multiple sources/scanners. With preliminary classification this produced 419 positive cases, which were used for collecting the training set. Due to the small number of preliminary positive cases, another randomly sampled cohort consisting of preliminary positive cases was selected for the test set, excluding patients that were in the first set. This produced 598 cases. The first search produced 2329 preliminary negative cases, which was enough for both the training and test sets.

We chose to weakly annotate imaging data by not labelling distinct emboli with bounding boxes or segmentations, but rather assigning binary labels (PE positive or PE negative) only to axial slices and whole CT scans. The rationale was to both minimize manual work and test whether weakly annotated data suffice for a DL network to achieve reasonable results.

For this study, only CTPAs with readily available 3.0 mm axial slices were included. Scans with non-diagnostic image quality were excluded. All CTPAs were visually interpreted, and a scan was manually labelled positive if it included even one unambiguous embolus and negative if there were none. A slice was marked positive if one or more emboli were present, and negative if none were present. In the training set, the labelling work was done by a radiology resident (H.H, < 1 year of experience) specifically trained for this task by an experienced board-certified radiologist (M.N., 14 years of experience). In the test set, all scans and slices were double read by H.H. and M.N. in consensus to achieve a better reference standard. Interpretation and labelling were done using Horos software (Horos Project, Annapolis, MD, USA) and ePad annotation platform (Rubin Lab, Stanford Medicine, CA, USA). Collecting and labelling the data was performed securely on PACS inside the hospital network, and the data was de-identified before moving it to the computing platform.

For the training and test sets, 303 and 97 positive CTPAs were collected, respectively. To achieve balanced datasets, equal numbers of negative CTPAs were selected randomly for the training set (305) and test set (107). Because some of the CT studies extended cranio-caudally to the level of abdomen and pelvis, the slice coverage was limited to only 96 slices starting from the top (96 × 3 mm = 288 mm). This was deemed sufficient, because all positive slices in the training set fell within this range. The training set had a total of 52,752 included slices, of which 7170 were positive (14%), and the test had a total of 17,778 included slices, of which 2801 were positive (16%) (Table 1). The datasets did not include the same patients. No systematic differences were observed between the training and test sets in terms of patient demographics, type of scanner used, or the time period the scans were acquired.

**Table 1:** Dataset information

	Training set	Test set
CTPAs (stacks)	608	204
Positive	303 (50%)	97 (48%)
Negative	305 (50%)	107 (52%)
CT slices	52,752	17,778
Positive	7170 (14%)	2801 (16%)
Negative	45,582 (86%)	14,977 (84%)
Distinct patients	569	201
Male	250 (44%)	88 (44%)
Female	319 (56%)	113 (56%)
Mean age	64	64
CT manufacturer		
Toshiba	569 (94%)	195 (96%)
GE Medical Systems	21 (3%)	5 (2%)
Siemens	18 (3%)	4 (2%)

### Data augmentation and preprocessing

We augmented data to increase the number of positive slices in the training set, because the ratio between positive and negative slices was highly imbalanced. For augmentation, we used methods including translation, rotation, blur, Gaussian noise, zoom and elastic transformation. The final training set for the CNN models consisted of ~ 100,000 slices, of which half were negative and half were positive (the original 7170 real positive slices and ~ 43,000 augmented positive slices). Augmented data was not used in training the LSTM part, because it handled the data on the stack-based level which was already balanced between the positive and negative classes.

Preprocessing of the images included rescaling, resizing, segmentation and windowing. Images had originally a height and a width of 512 pixels, except for 10 scans where the original width was larger, and rescaling had to be applied. Images were resized to 386 × 386 pixels, which was the input size required by the CNN model. Segmentation was performed to reduce the amount of unnecessary information in the images and was done by segmenting the lungs and the heart and excluding other areas (e.g., soft tissues and objects outside the body). Images were windowed with window width 700 HU and window level 100 HU. Finally, DICOM images were converted to 8-bit PNG images.

### Model architecture and training

All models were created using Keras deep learning framework (version 2.2.4) with Tensorflow backend (version 1.10.1) and were written in Python programming language (version 3.6, Python Software Foundation).

**Fig. 1:**
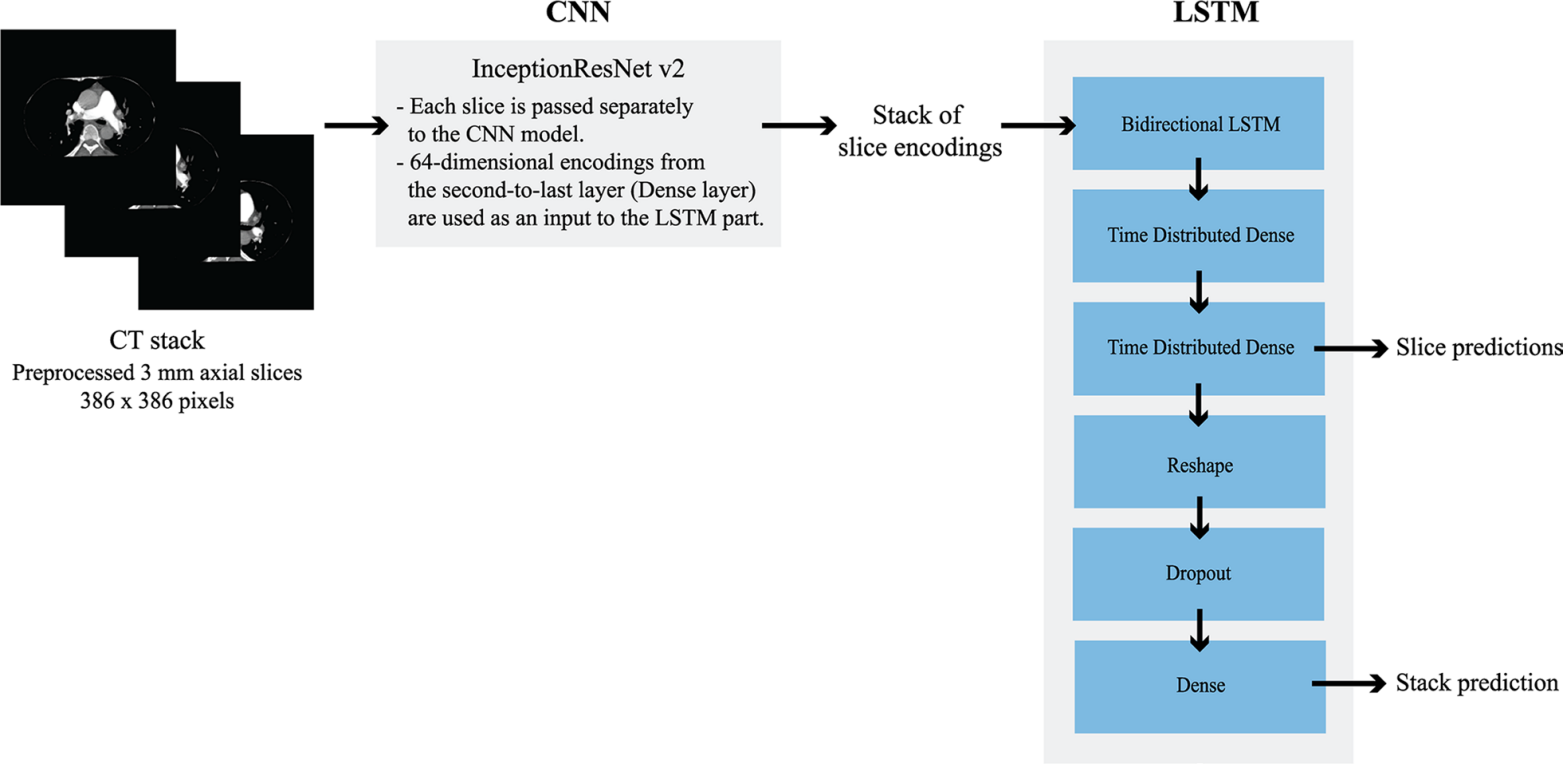
Scheme of the model architecture

We processed whole CT scans as series of axial slices using a combination of a CNN, which analyses 2D images, and an LSTM, which processes the slice predictions created by the CNN part as sequences (Fig. 1). A major advantage in this approach is that 2D CNN models are considerably easier to train and require less data than 3D CNN models. The LSTM part also allows the model to take findings in neighbouring slices into consideration.

Based on our preliminary experiments with different pre-trained CNN model architectures, we selected InceptionResNetV2 to be used in this study [33]. A simple custom classifier was built on top of the model bottleneck. As a secondary step, a bidirectional LSTM processed data from the CNN model creating a final combination model. Detailed model architectures can be found in the Additional file 1. Transfer learning was implemented, and two separate combination models were trained with different pre-trained weights for the CNN part. Model A was pre-trained with the chest X-ray dataset provided by National Institutes of Health (over 100,000 chest X-rays, weights obtained from https://github.com/i-pan/kaggle-rsna18) and Model B was pre-trained with the ImageNet dataset (over 14 million natural images). The LSTM used 64-dimensional feature vectors from the second to last layer of the CNN as an input. We chose to pre-calculate these feature vectors and train the LSTM part independently from the CNN, because training them together would have required more time and more laborious adjustments of the hyperparameters. A fixed timestep of 96 slices starting from the top was used. CTPAs containing more than 96 slices were clipped and the CTPAs having less than 96 slices were padded with zeros.

All models were trained using a five-fold cross-validation. In this method, the training data is split into five folds of equal size, and five “copies” of the model are trained, each time using a different fold as a validation set to evaluate training results. This method gives a more truthful estimation of the model accuracy when datasets are relatively small. The folds were stratified so that each fold maintained the same distribution of both the class and the number of augmented images as the whole training set, and images from a given patient were always kept in the same fold. During training, augmented images were omitted from the validation set, which then had the original ratio of negative to positive slices (7:1) and CTPAs (1:1).

For the CNN and LSTM models, batch sizes were 48 and 16, and the numbers of epochs were 8 and 12, respectively. Binary cross-entropy was used as a loss function. The optimizers used were Adam with a decay value of 0.01 for CNN models and Nadam for LSTM models. Learning rate was 0.001 for all models. The models were trained using 4 NVidia V100 SXM2 32 GB graphics cards.

### Performance evaluation and statistical analysis

The final combination models A and B produced both stack-based and slice-based predictions, and their performance was evaluated on both levels. We plotted receiver operating characteristic (ROC) and precision–recall (PR) curves for test results and calculated their area under the curve (AUC) values. We compared ROC curves of Model A and B using the DeLong method [34]. Statistical significance was set at *p* < 0.05. We determined the best operating thresholds according to the Youden Index, which gives equal weights to sensitivity and specificity as well as to positive and negative classes. Predictions above this threshold were classified as positive and otherwise as negative. Using selected operating thresholds, we calculated confusion matrices for both stack-based and slice-based predictions. We evaluated the model performance based on five metrics, consisting of accuracy, sensitivity, specificity, positive predictive value (PPV) and negative predictive value (NPV), and calculated their 95% confidence intervals (CI) using the Wilson test with continuity correction [35]. We also visually inspected example images of true positives (TP), true negatives (TN), false positives (FP) and false negatives (FN). Model testing was performed using Python programming language (version 3.6, Python Software Foundation). Calculations and statistical analyses were performed using Python, R (version 4.1.0), and RStudio (version 1.4.1717).

## Results

**Fig. 2:**
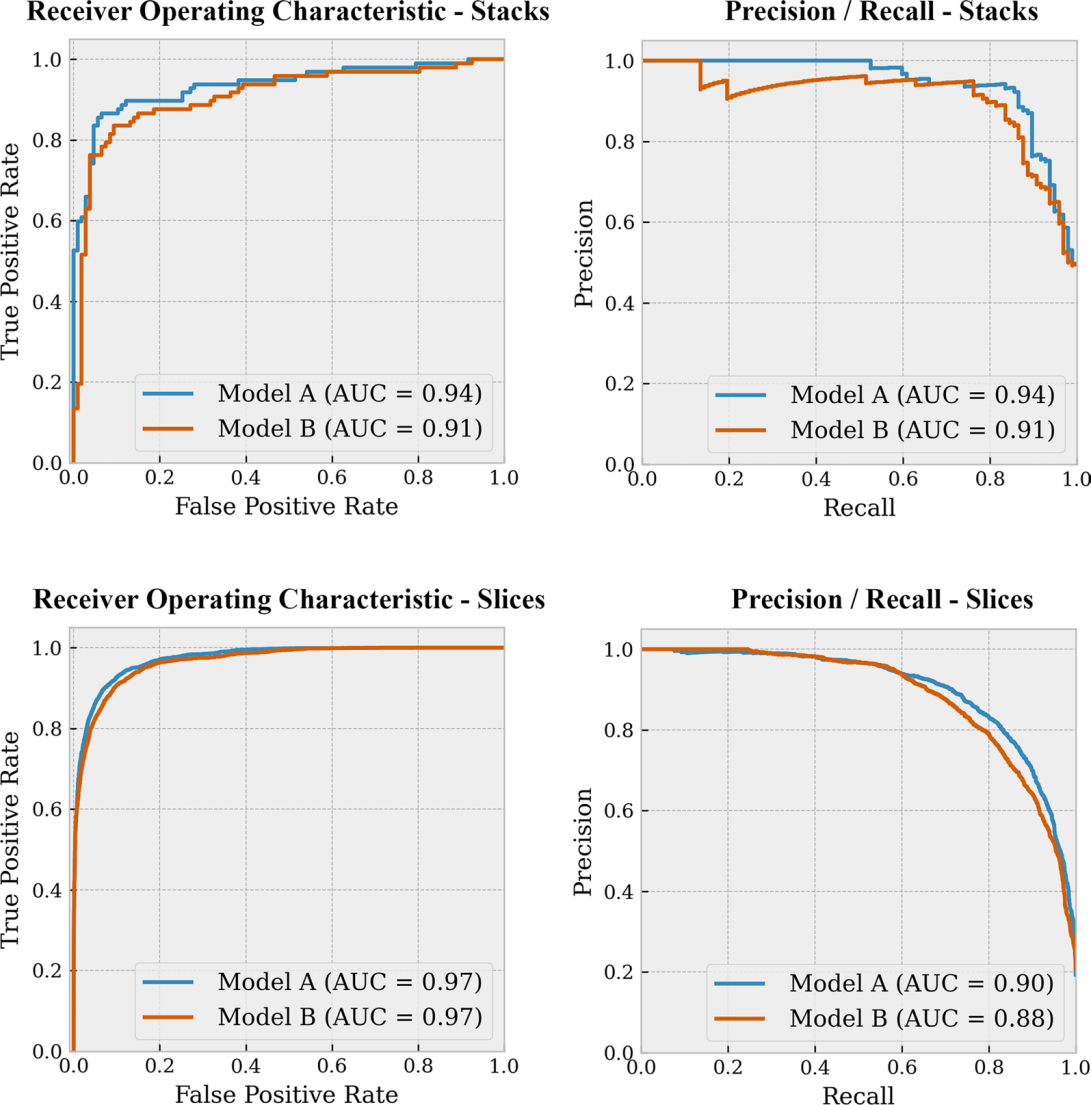
ROC and PR curves for stack-based and slice-based predictions

Both combination models achieved excellent results on the test set. For Model A, ROC AUC and PR AUC scores for predicting PE on the stack-based level were 0.94 and 0.94, and on the lice-based level 0.97 and 0.90, respectively. For Model B, ROC AUC and PR AUC scores on the stack-based level were 0.91 and 0.91, and on the slice-based level 0.97 and 0.88, respectively. The ROC and PR curves for both models are represented in Fig. 2. ROC curve comparisons between Models A and B showed that there was no statistically significant difference on the stack-based level (*p* = 0.07). On the slice-based level, there was a significant difference (*p* < 0.001), but this seems very minimal as the ROC curves and AUC values are almost identical.

The optimal thresholds for classifying the predictions determined by the Youden index were similar between the models, but different between the stack-based and slice-based levels. On the stack-based level, the optimal Youden indices for models A and B were 0.80 and 0.74, corresponding to thresholds 0.797 and 0.858, respectively. On the slice-based level, the Youden indices were 0.83 and 0.81, corresponding to thresholds 0.172 and 0.094, respectively.

**Fig. 3:**
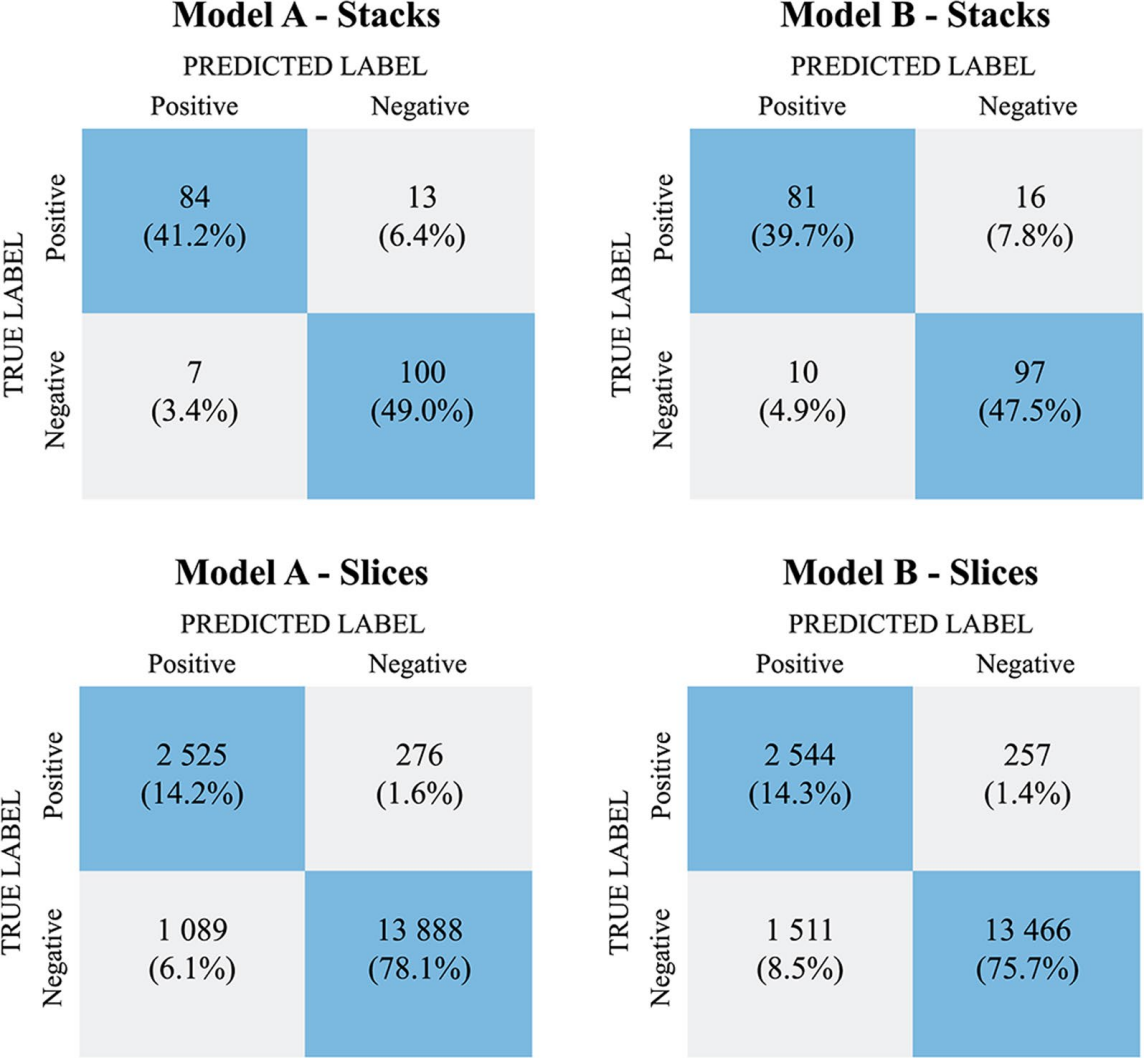
Confusion matrices for Models A and B on stack- and slice-based classification

Confusion matrices calculated using these thresholds are shown in Fig. 3.

The performance metrics for models on both stack- and slice-based predictions are represented in Table 2. Model A outperformed Model B on all metrics on the stack-based level. The accuracy, sensitivity, and specificity of Model A were 90.2%, 86.6% and 93.5%, respectively. On the slice-based level, both models also had good performance but their PPV was considerably lower than on the stack-based level (Model A 69.9% vs. 92.3%; Model B 62.3% vs. 89.0%, respectively).

**Table 2:** Performance metrics for Models A and B

Metric	Model A		Model B	
	Stacks	Slices	Stacks	Slices
Accuracy	90.2 (85.1–93.8)	92.3 (91.9–92.7)	87.3 (81.7–91.4)	90.1 (89.6–90.5)
Sensitivity	86.6 (77.8–92.4)	90.1 (89.0–91.2)	83.5 (74.3–90.0)	90.8 (89.7–91.9)
Specificity	93.5 (86.5–97.1)	92.7 (92.2–93.1)	90.7 (83.1–95.2)	89.9 (89.4–90.4)
PPV	92.3 (84.3–96.6)	69.9 (68.3–71.4)	89.0 (80.3–94.3)	62.3 (61.2–64.2)
NPV	88.5 (80.8–93.5)	98.1 (97.8–98.3)	85.8 (77.8–91.4)	98.1 (97.9–98.3)

Results are in percentage (%) with 95% CI

**Fig. 4:**
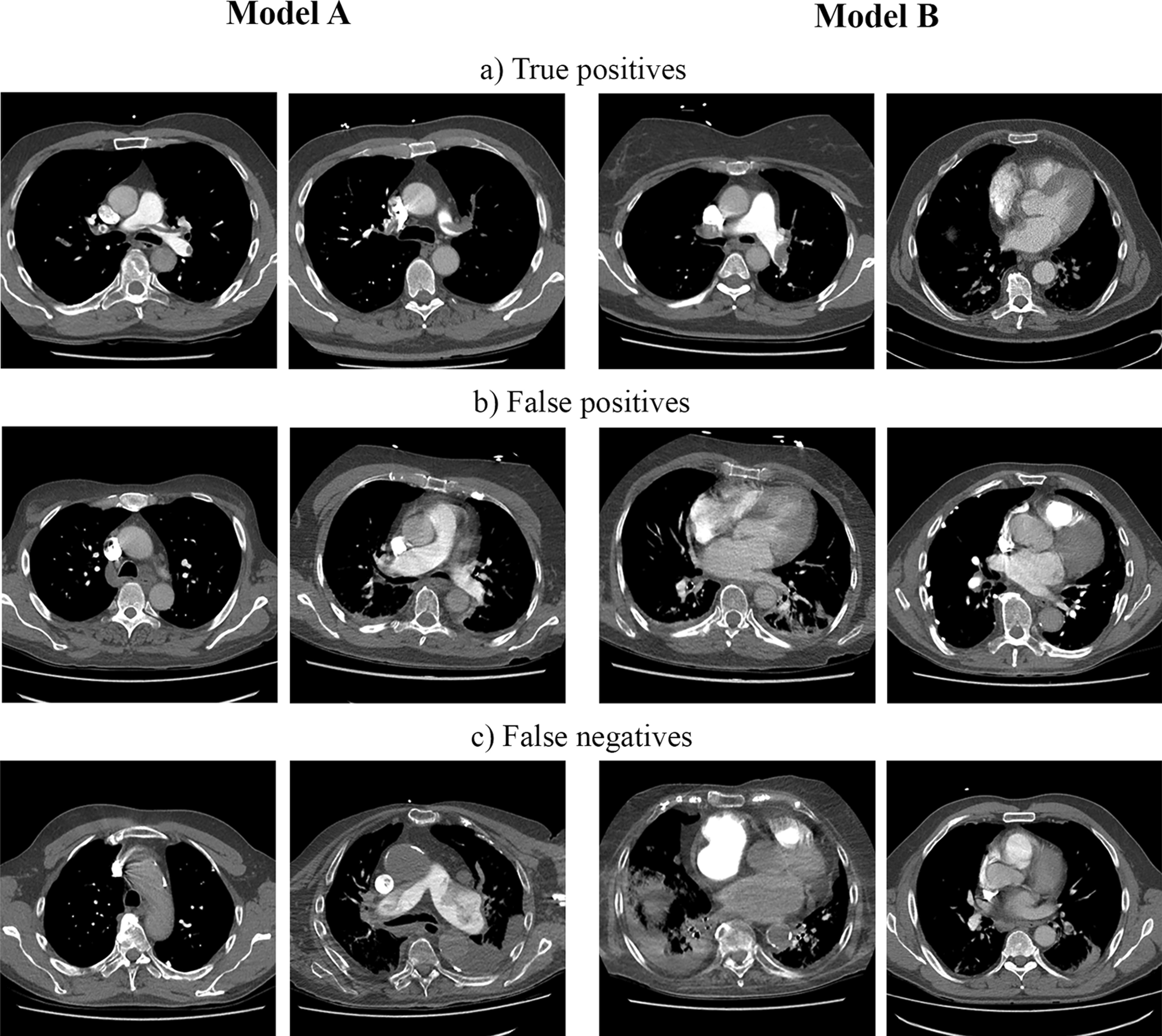
The figure shows example slices where both the slice-based and stack-based predictions are **a** true positives, **b** false positives or **c** false negatives. In the left panel (the first two images on each row from left to right) are images classified by Model A and in the right panel (the last two images on each row) are images classified by Model B

Example images from true positive, false positive and false negative classifications for both models were visually inspected (Fig. 4). We did not notice any major differences between the models. True positives seemed to be more often large proximal emboli, and false findings were more often located in the peripheral parts of the pulmonary arteries. This was expected, because small and peripheral emboli are usually difficult also for radiologists.

## Discussion

The main contribution of this study is to demonstrate the development of a deep learning model for automated detection of PE from CTPAs, and to show that this is feasible even with limited data annotation resources. We found that our best model (Model A) achieved an ROC AUC of 0.94, sensitivity of 86.6% and specificity of 93.5% in predicting PE from whole CTPA stacks. We showed that these promising results could be achieved using a weakly labelled training dataset consisting of only 600 CTPAs, which is a relatively small dataset for neural networks.

Our models used less training data, yet performed better than several models presented in previous studies [26, 28, 36]. Our best model also had better specificity with almost similar sensitivity than a multimodal fusion model combining information from CT images and electronic health records, which had a specificity of 90.2% and a sensitivity of 87.3% [37]. We achieved only slightly worse specificity with our best model than Aidoc’s commercial model (93.5% vs. 95–95.5%, respectively), despite their model having been trained with a considerably larger dataset (600 vs. ~ 28,000 CTPAs) [24, 38]. Models presented by Yang et al. and Tajbaksh et al. aimed to localize each distinct embolus accurately, and their performance was evaluated differently than our model, which is why the models cannot be directly compared [21, 39].

Transfer learning and data augmentation were implemented to compensate for the small training set size. The choice of transfer learning data had only minimal impact on the model performance. Model A performed as well as or slightly better than Model B, even though it was pre-trained on a substantially smaller dataset than Model B (NIH dataset, 100,000 images vs. ImageNet dataset, 14 million images). Since the ImageNet dataset consists of color images, the model pre-trained with it learns many features irrelevant to classifying grayscale CTPA images. NIH chest X-rays are grayscale like target data, and therefore a smaller dataset seems to suffice.

Compared to many previous studies, our approach to annotate the data with only stack- and slice-based binary labels was more time-saving than marking each distinct embolus [15, 21, 39]. Rajan et al. as well as Shi et al. reduced the manual workload in annotation by doing pixel-wise segmentations only on slices at 10 mm intervals and otherwise using CT study-level binary labels [26, 36]. Despite using sparser annotations, our model performed better than their models. Huang et al. used data which was annotated in a similar fashion than in our study [28]. However, they excluded studies with only subsegmental emboli due to their unclear clinical importance, but we felt the need to include them because they represent a substantial part of the realistic data seen in clinical practice.

There was a notable difference between stack and slice level cut-off thresholds selected with the Youden index that needs to be discussed. The Youden index is a measurement for the ROC curve and it is used to select the optimal operating point on the curve. The optimal cut-off threshold corresponding to this operating point depends on the range and distribution of the model output scores, which can vary wildly between models. Without additional calibration, the model output score does not always equal the probability of the predicted class [40]. Also, another reason why the thresholds between the stack and slice levels are not comparable is that the positive class ratios were very different between the levels (48% vs. 16%, respectively).

Our study had certain limitations. First, our models do not produce precise information about the location of emboli, which might hinder the interpretation of model findings. Including localization of emboli in our model would have increased the manual annotation workload considerably. Our model can still pinpoint individual slices that are most likely to contain PE to help radiologists quickly focus on the most probable area. Exact localization of emboli is also not needed in pre-screening CT studies to prioritize the reading list. Our model could help in faster detection of PE positive cases in emergency settings as well as in non-emergency CT imaging where an incidental PE is frequently found, especially in oncologic patients, where there might be a several days delay in reading the CT scans [41, 42]. Second, we only used data from our institution, and the majority of the data was acquired with CT scanners from one vendor. Our model might need further training to perform accurately with data acquired in different institutions or with different CT scanners, as model performance often degrades on data acquired differently (e.g., different CT scanner model, manufacturer or imaging settings) [43]. Third, our model was tested only on a dataset balanced between positive and negative cases. In the clinical setting, the prevalence of positive CTPAS is much lower, varying from less than 10% to 30% [9]. Using balanced datasets is, however, a common practice in medical AI research. In the future, we aim to test the model on a dataset representing a more realistic prevalence.

## Conclusions

In conclusion, we developed a deep learning model for automated PE detection which achieved substantial performance results. We showed that these results could be achieved with a relatively small, weakly labelled training set. This demonstrates that it is possible for small research groups and individual hospitals to build well-performing DL models even with limited resources. Our model could be used either as an aid in reading emergency studies to reduce mistakes, or as a pre-screening triage tool to prioritize reading order. In the future, we plan to test the model performance and usability in a clinical setting. We plan to study how the model performs when data acquisition is slightly modified (e.g., updated scanning protocol, or a new scanner), and how much additional training the model requires after each change. We also plan to develop the visualization of the output for better interpretability.

## Supplementary Information

**Additional file 1.** Model architectures. Detailed model architectures for the CNN model and the CNN + LSTM combination model.

## Data Availability

Image data cannot be publicly shared because of the national legislature on patient data. The source code for image preprocessing and training the models will be published on GitHub (https://github.com/turku-rad-ai/pe-detection) by 31st of March 2022. Otherwise all relevant data are within the manuscript and its supplementary information. Further inquiries should be addressed to Jussi Hirvonen (jussi.hirvonen@utu.fi).

## Abbreviations

PE: Pulmonary embolism
CTPA: Computed tomography pulmonary angiogram
CT: Computed tomography
DL: Deep learning
AI: Artificial intelligence
CAD: Computer-aided detection
CNN: Convolutional neural network
LSTM: Long-short term memory network
FP: False positive
FN: False negative
TP: True positive
TN: True negative
ROC: Receiver operating characteristics
PR: Precision–recall
AUC: Area under the curve
PPV: Positive predictive value
NPV: Negative predictive value
CI: Confidence interval
