# Supplementary figures and images for: Automated detection of pulmonary embolism from CT-angiograms using deep learning
Source: BMC Med Imaging. 2022 Mar 14;22:43. doi: 10.1186/s12880-022-00763-z (PMC8919639; doi:10.1186/s12880-022-00763-z)

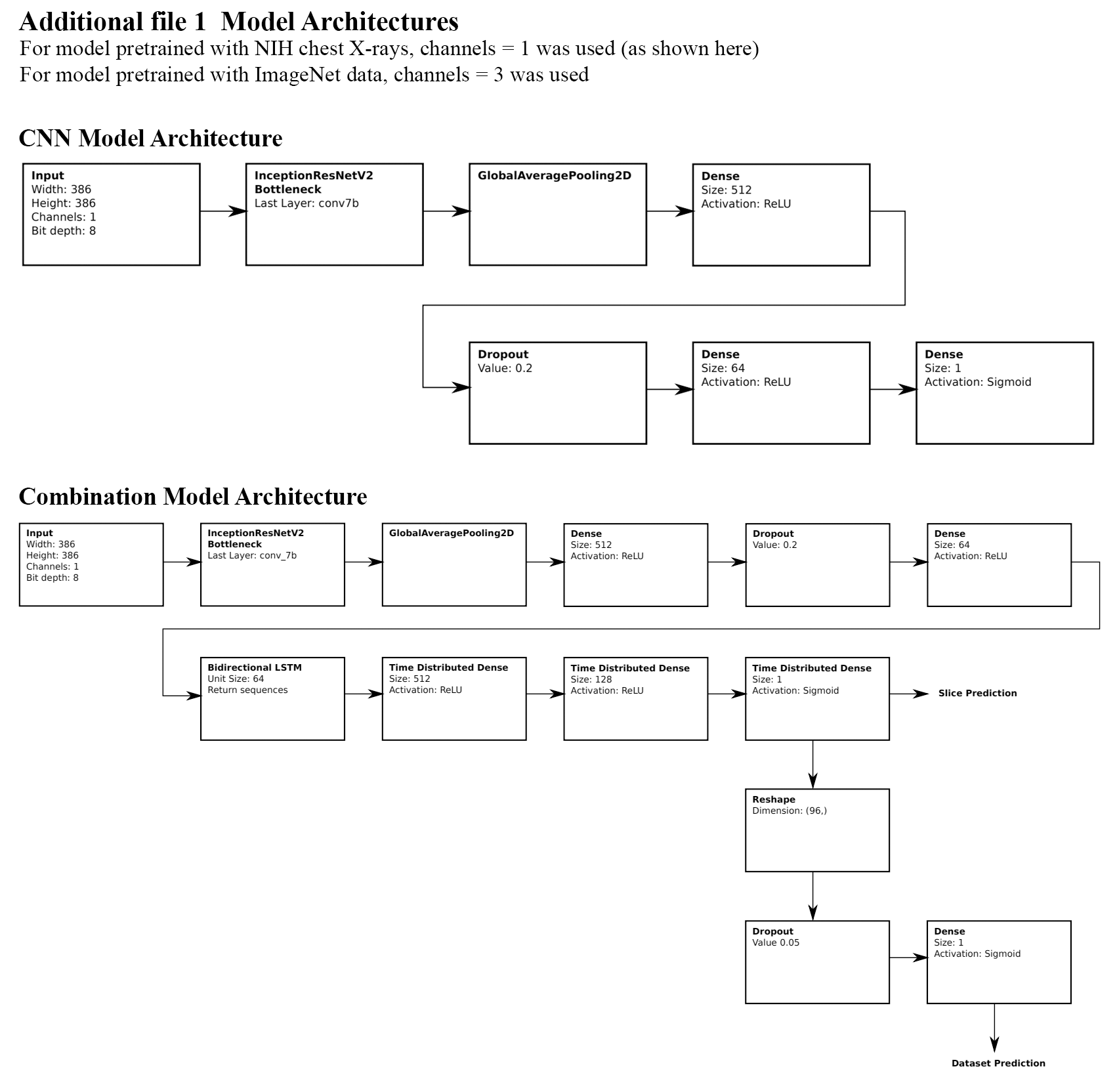

Supplement: Supplementary file 1 — Additional file 1. Model architectures. Detailed model architectures for the CNN model and the CNN + LSTM combination model. [file 12880_2022_763_MOESM1_ESM.tif]
